# Burden of disease resulting from lead exposure at toxic waste sites in Argentina, Mexico and Uruguay

**DOI:** 10.1186/s12940-016-0151-y

**Published:** 2016-06-23

**Authors:** Jack Caravanos, Jonathan Carrelli, Russell Dowling, Brian Pavilonis, Bret Ericson, Richard Fuller

**Affiliations:** School of Public Health, City University of New York, 2180 Third Ave., New York, NY 10035 USA; Pure Earth, formerly Blacksmith Institute, 475 Riverside Drive, Suite 860, New York, NY 10115 USA

**Keywords:** Latin America, Burden of disease, Disability-adjusted life year, Chemical exposure, Toxic waste sites, Lead poisoning

## Abstract

**Background:**

Though lead contaminated waste sites have been widely researched in many high-income countries, their prevalence and associated health outcomes have not been well documented in low- and middle-income countries.

**Methods:**

Using the well-established health metric disability-adjusted life year (DALY) and an exposure assessment method developed by Chatham-Stephens et al., we estimated the burden of disease resulting from exposure to lead at toxic waste sites in three Latin American countries in 2012: Argentina, Mexico and Uruguay. Toxic waste sites identified through Pure Earth’s Toxic Sites Identification Program (TSIP) were screened for lead in both biological and environmental sample media. Estimates of cardiovascular disease incidence and other outcomes resulting from exposure to lead were utilized to estimate DALYs for each population at risk.

**Results:**

Approximately 316,703 persons in three countries were at risk of exposure to pollutants at 129 unique sites identified through the TSIP database. Exposure to lead was estimated to result in between 51,432 and 115,042 DALYs, depending on the weighting factor used. The estimated burden of disease caused by exposure to lead in this analysis is comparable to that estimated for Parkinson’s disease and bladder cancer in these countries.

**Conclusions:**

Lead continues to pose a significant public health risk in Argentina, Mexico, and Uruguay. The burden of disease in these three countries is comparable with other widely recognized public health challenges. Knowledge of the relatively high number of DALYs associated with lead exposure may be used to generate support and funding for the remediation of toxic waste sites in these countries and others.

## Background

Environmental exposure to pollution from hazardous waste sites is an understudied contributor to the global burden of disease [1]. Increasing industrial development, urbanization and socioeconomic forces in Latin America have contributed to an increase in environmental pollution and the negative health effects resulting from exposure [2]. The disability-adjusted life year (DALY), which takes into consideration the burden of disease resulting from illness, injury and death, is a standard metric for estimating the burden of disease resulting from exposure to environmental toxicants, among other risk factors. A previous study by Prüss-Ustün estimated that exposure to various chemicals accounts for 5.7 % of total global DALYs and 8.3 % of global deaths [3]. Another study estimated that 0.22 % of the total estimated DALYs from all causes were attributed to pollutants found at hazardous waste sites in India, Indonesia and the Philippines [4].

It is estimated that 94 % of the burden of disease resulting from pollution falls on low- and middle-income countries as defined by the World Bank (LMICs) [5]. While much of the developed world has made significant progress in eliminating the burden of disease caused by infectious diseases, chronic illnesses increasingly affect a great population [6]. Chronic illnesses, such as cardiovascular disease, neurodevelopmental disorders and cancers are often linked to environmental exposures, yet enumerating the specific burden of disease impacts from environmental agents has proven difficult [7].

There is a need to better understand linkages between contaminated sites and health outcomes in LMICs. Accurate DALY models enumerated by contaminant, exposure pathway, and affected population offer one possible approach. Summary measures may then be used during the policymaking process to discern what public health threats are of greatest concern and what policies are most effective [8].

Heavy metals are still widely used in the production of consumer goods [9]. In LMICs, inadequate regulation, informality of many industries, poor surveillance, and improper disposal of contaminants can result in dangerous exposures to nearby residents. Of particular concern is lead (Pb). Although the risk of disease resulting from exposure to lead is widely known, widespread use continues [10]. For example, lead is still used to glaze artisanal ceramics despite the availability of less hazardous alternatives. Elevated levels of lead in water and soil can then expose adjacent populations, putting them at risk of a number of adverse health outcomes.

Lead is a bluish-gray metal with many desirable qualities such as electrical conductivity, malleability, density and low-corrosivity and has been mined for centuries, often combined with other metals to form alloys [10]. Anthropogenic sources of lead in the environment include smelting, mining, used lead-acid battery (ULAB) recycling and ceramic pottery making [11, 12]. Compared to adults, children absorb more lead and are therefore more vulnerable to the adverse effects of lead. Early childhood exposure to lead can be particularly harmful and has been shown to cause behavioral problems in adolescence [13], IQ decrements [14], cognitive impairment [15], and decreased visuospatial skills [16]. Adults are typically exposed occupationally and experience higher rates of hypertension than the general population, leading to an increased incidence of cardiovascular disease [17]. Low-level chronic exposure to lead may result in low sperm count or impotence in males. In females, it can result in miscarriage and low birth weight of offspring, as lead may be transferred through the placenta to the fetus [16].

This research aims to accurately quantify the burden of disease caused by lead found at toxic waste sites in Argentina, Mexico and Uruguay. Earlier work done by Caravanos et al. described the pediatric burden of Pb and other heavy metals exposure in several Asian countries [18]. This analysis seeks to elucidate the impact of lead on human health in a different part of the world. The resulting analysis aims to provide a basis for public health intervention and environmental remediation at both the national and regional level, as well as to inform strategies for continued site investigation of contaminated sites.

## Methods

### Site identification

Environmental and biological exposure data were obtained from the Toxic Sites Identification Program (TSIP). The TSIP is an effort implemented by the New York-based non-profit Pure Earth (formerly Blacksmith Institute) and has been supported by the United Nations Industrial Development Organization (UNIDO), the European Commission (EC), the World Bank, and the Asian Development Bank, among others. The TSIP identifies active and abandoned hazardous waste sites resulting from both formal and informal industrial activities in LMICs. Informal activities include but are not limited to electronic waste or scrap metal recycling, used lead-acid battery recycling, small-scale gold mining, leather tanning, and ceramic pottery making. There are currently more than 3200 sites in the TSIP database, of which 2300 have been visited onsite by a trained TSIP investigator. A majority of the locations screened are abandoned (legacy) sites, including former tanneries and small-scale artisanal sites such as ULAB recycling and artisanal gold mining [4]. The TSIP does not include exposure data from non-point sources such as vehicle traffic or sewage contaminated water. As part of a TSIP investigation, a “key pollutant” is identified and analyzed. Heavy metals are the most commonly occurring key pollutant, with ingestion of contaminated soils being the most commonly occurring route of exposure listed in the TSIP database [19].

Three countries were utilized for this analysis: Argentina, Mexico and Uruguay (Table 1). These countries were chosen primarily on the basis of the availability of data after the inclusion and exclusion criteria outlined below were applied to the raw data. Sites that did not meet all five criteria were not included in the analysis. No additional data collection was conducted for this paper.

**Table 1:** Country demographics^a^

Country	Total population (in millions)	Population density (inhabitants per km^2)^	GDP per capita (USD)	Infant mortality (per 1,000)	Life Expectancy (years)
Argentina	41.45	14.4	22375	11	76.01
Mexico	122.3	57	10174	11	77.14
Uruguay	3.4	18.9	16996	9	76.91

^a^The World Bank, 2014

In order for a hazardous waste site to be included in the analysis, five criteria must have been met: there must be a credible pathway of human exposure; a biological or environmental sample had to be present; a population at risk had to be specified; the location of the site was represented by GPS coordinates; and a description of the activities leading to contamination were outlined. The TSIP database contained 23 site surveys in Argentina, 62 in Mexico, and 44 in Uruguay that met the inclusion criteria of this study. A total of 129 sites analyzed with data from 164 environmental lead samples and 75 blood lead level measurements were included in the analysis. It should be noted that while numerous sites contain both blood lead and soil lead data, there are also many sites that contain data from only one sample medium.

### Exposure assessment

Local site investigators in the field collected environmental samples with the guidance of a sampling protocol provided by Pure Earth. Biological samples were made available through collections by local health offices and ministries. An independent ethics committee determined that the study was exempt from further review as categorized by the US Department of Health and Human Services Policy for Protection of Human Research Subjects. Lead concentrations in soil were measured in the field using an Innov-X handheld X-ray fluorescence (XRF) spectrometer (4000 Alpha Series; Auburndale/Newton, MA). XRFs are calibrated accordingly prior to soil sample analysis. When an XRF was unavailable, samples were sent to a local laboratory for analysis. Exposure pathways in the analysis included inhalation of dust and ingestion of lead contaminated soil. All Pb exposure was estimated through blood lead levels (BLLs) (*n* = 75) or soil concentrations (*n* = 164). In areas suspected of lead contamination, BLLs were prioritized, as they are the standard marker of human exposure [20]. The U.S. Centers for Disease Control and Prevention (CDC) sets an upper limit of 5 μg/dL for children under the age of 6 years [21]. While the “actionable” reference BLL was lowered from 10 μg/dL to 5 μg/dL in 2012 by the CDC, 10 μg/dL is still the standard reference BLL in most countries [22]. When a site contained less than 5 biological samples, environmental sample data such as lead in soil was used to calculate burden of disease estimates.

### Population estimates and age distribution

An age distribution of the population must be used when calculating the burden of disease*.* It has been well documented that children are more susceptible to negative health effects caused by exposure to toxic pollution than adults [23, 24]. Hazardous chemicals are ingested and inhaled into the body of children at a much higher rate than in adults. Furthermore, toxicants can affect children during critical windows of development when children’s bodies and neurological function are most at risk [24]. Children also engage in more high-risk behaviors when compared to adults—they are lower to the ground and tend to have more unwashed hand to mouth contact [18]. As age distribution was not recorded as part of TSIP protocol, province-specific age distributions from the respective countries’ census institutions were used in disease estimates [4, 25–28].

A local country specific investigator develops a Conceptual Site Model (CSM) for each site assessed as part of the TSIP. The CSM allows the investigator to determine key sources, migration routes, and chemical exposure pathways. Additionally the investigator using the CSM determines the estimated “population at risk.” A population count is then generated from residences and communities adjacent to all sources of exposure using reported housing densities (number of persons per household). High-resolution aerial imagery is also used to confirm population estimates by reviewing the number of people residing within the affected area (defined as having a radius of 50 m). For the purpose of this analysis, population at risk estimates were reviewed against similar sites in the TSIP database.

### Risk estimates

Risk was calculated for non-carcinogenic health endpoints based on lead toxicity [4]. Disease incidence and burden for lead were calculated separately using the USEPA’s Integrated Exposure, Uptake and Biokinetic (IEUBK) model and tools developed by the World Health Organization (WHO) [29, 30]. The IEUBK model is used to estimate BLLs in children resulting from Pb exposure via soil, air, water, food, and maternal blood lead [20].

The IEUBK Model is a validated tool that estimates the geometric mean of BLL from exposure to multiple sources of lead. However, for this analysis we limited the model to soil lead exposure from each site. We entered these values into the model and calculated mean BLLs for each site. Exposure intakes for air lead levels, dietary intake of lead, water lead levels, maternal BLL and alternate sources of lead were set to “zero” so that the resultant estimated BLL is attributable solely to soil lead exposure. The IEUBK EPA model is specific to children so in estimating adult blood lead levels we applied the USEPA’s Adult Lead Methodology (ALM) exposure model [31]. As with IEUBK, only lead in soil inputs were used in the model with all other sources set to “zero”.

### Incidence of disease

Blood lead levels from exposure to environmental soil and dust lead levels were estimated using the US EPA’s IEUBK model. Exposure estimates were calibrated upward to account for the typically dustier conditions of low-income areas in LMICs. Values used elsewhere for indigenous populations were utilized here [32–34]. DALYs resulting from measured blood lead level samples and estimated blood lead levels were calculated separately using disease incidence and spreadsheets created by the WHO [35]. Using these spreadsheets, both incidence of mild mental retardation (MMR) in children and cardiovascular disease in adults were calculated for lead [4].

### Burden of disease calculation

The DALY is a time-based measure of health that combines indices of years lived with disability (YLD) and years of life lost (YLL). YLD and YLL were calculated based on exposure estimates collected in the field. YLD is the product of years lived with a disability and a specific disability weight (DW). A DW is scaled between zero and one, with zero representing perfect health and one representing the worst possible state of health (equivalent to death) [36]. For example, mild mental retardation attributable to lead exposure has a DW of 0.36 while metastatic lung cancer has a DW of 0.75 [37].

In order to calculate YLD the relevant type of non-cancer health effect was matched with the corresponding DW (i.e., neurological effects) [4]. Years lived with disability resulted from an estimation of life expectancy multiplied by the appropriate disability weight for the exposure scenario [4]. Years of life lost were calculated only for exposure to carcinogens; as a result, lead exposure did not contribute to YLL [4]. This is the standard method for calculating lead induced MMR, as lead exposure very rarely results in death.

DALYs resulting from cardiovascular disease were transformed into percentages to show a distribution across age groups for each country using the WHO’s Global Health Estimates Summary Tables [29]. The percentage of DALYs attributable to ischemic heart disease, cerebrovascular disease, hypertensive disease and all other cardiac diseases were calculated for each country individually. By using BLLs and this percentage of DALYs attributable to cardiovascular disease in a WHO spreadsheet, DALYs attributable to lead exposure were calculated [4, 38].

Age weighting factors, along with a discount rate, were applied to both YLD and YLL to provide a range of DALY estimates. Age weights are applied to burden of disease estimates in an effort to reflect the relative population distribution, while discount rates are often employed in burden of disease studies to account for intergenerational differences in health benefits reaped from public health interventions and a decrease disease incidence [4, 39]. Both the age weights and discount rates are signified in the notation DALYs_(r,K)_, where r is the discount rate and K is the age weight. Results expressed as DALYs_(3,1)_ represent a 3 % discount rate (recommended by the U.S. Panel on Cost-Effectiveness in Health and Medicine and utilized by the WHO) and full age weighting, while those expressed as DALYs_(3,0)_ include only the discount rate [40]. DALYs_(0,0)_ represent a burden of disease estimate without weighting.

### Sensitivity analysis

A range of estimates was also created through a sensitivity analysis by adjusting the size of the population at risk. This analysis calculates the effect of lead on a population plus and minus 25 % of the current estimate to account for possible fluctuations in the population.

## Results

**Fig. 1:**
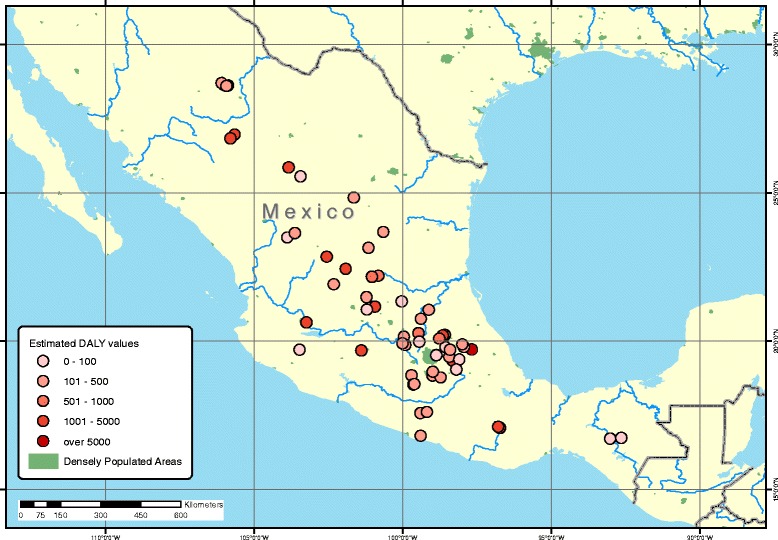
Geographical distribution of TSIP sites in Mexico with DALYs resulting from lead exposure

**Fig. 2:**
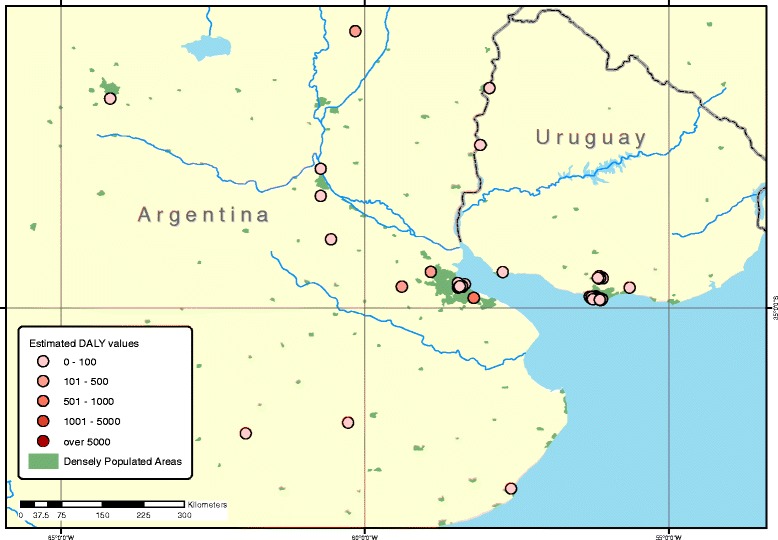
Geographical distribution of TSIP sites in Argentina and Uruguay with DALYs resulting from lead exposure

Exposure data were collected from a total of 129 hazardous waste sites distributed across Argentina (*n* = 23), Mexico (*n* = 62), and Uruguay (*n* = 44). The geographical distributions of these sites are shown in Figs. 1 and 2. The estimated population at risk of exposure was 316,703 individuals (mean = 2455; median = 250 per site), which is approximately 0.19 % of the total population of all three countries. Of this population, it was estimated that 80,021 were women of childbearing age (15–49 years of age), and 122,084 individuals were younger than 18 years of age (Table 2). Of the exposed population, the proportion of women of childbearing age was relatively equal across the three countries.

**Table 2:** Exposed population by age and childbearing status

Country	Total Exposed Population (Population)	Women of Childbearing Age - 15–49 years old (Population)	All Genders <18 Years old (Population)	Remaining Age Groups (Population)
Argentina	112208	27852	36659	47697
Mexico	189593	48770	80904	59919
Uruguay	14902	3399	4521	6982
Total	316703	80021	122084	114598

Biological (*n* = 75) and environmental measurements (*n* = 164) were used to calculate risk. An arithmetic mean was calculated for environmental or biological samples at each site, unless the site’s test results differed more than one order of magnitude. In these cases, a geometric mean was used as outlined in the sampling protocol provided by Pure Earth. Mean blood lead levels (BLLs) in Mexico (*n* = 56) and Uruguay (*n* = 19) were found to be 19.63 μg/dL and 13.3 μg/dL respectively (Table 3). Lead concentrations in soil (*n* = 164) were the highest and relatively uniform across the three countries (Table 4). Mean soil lead concentrations in Mexico (2748 mg/kg) were the greatest, followed by Uruguay (2559 mg/kg) and Argentina (1730 mg/kg).

**Table 3:** Blood Lead Level (BLL) Data by Country

Country	N	Mean (μg/dL)	S.D. (μg/dL)	Range
Argentina	N/A	N/A	N/A	N/A
Mexico	56	19.63	9.9	7.7–41.1
Uruguay	19	13.3	9.21	5.1–47.35

**Table 4:** Environmental Lead Sample Data

	Argentina		Mexico		Uruguay	
	N	Mean^a^	N	Mean^a^	N	Mean^a^
Soil Samples (mg/kg)	48	1730	59	2748	57	2559

^a^Value shown is either the mean or single measurement

BLLs were used to estimate DALYs in exposed populations in Mexico (79,196) and Uruguay (5859). As BLLs were not collected in Argentina, DALYs based on that exposure measurement could not be calculated. Elevated BLLs were responsible for 23,421 DALYs in Mexico, representing 52 % of the disease burden estimated as a result of lead exposure. Elevated BLLs were responsible for 942 DALYs in Uruguay, representing 46 % of the total disease burden for lead exposure.

An estimated 27,069 DALYs resulted from exposure to lead in soil. Combined with an estimated 24,363 DALYs based on BLL, overall lead exposure accounted for a total of 51,432 YLDs. The estimated population at risk for exposure to lead was 316,703, largely derived in sites from Mexico (189,593) and Argentina (112,208). An estimated 0.31 DALYs_(3,1)_ per person resulted from lead exposure at 129 unique toxic waste sites screened in Argentina, Mexico and Uruguay.

Without age weights, 45,492 DALYs _(3,0)_ resulted, while removing both age weight and discount rate resulted in 115,042 DALYs _(0,0)_. To present a range of estimates, exposed population was adjusted to 25 % less than the original estimate, resulting in 38,581 DALYs_(3,1)_. If the exposed population was adjusted to 25 % greater than the original estimate, the resulting DALYs_(3,1)_ were 64,266. A remediation scenario where lead levels were adjusted below international standards resulted in 7,078 DALYs_(3,1)_ (Table 5).

**Table 5:** Sensitivity analysis estimates

Scenario	Total DALYs
Primary estimate of screened sites	51432 DALYs_(3,1)_
Estimate without age weights	45492 DALYs_(3,0)_
Estimate without age weights or discount rate	115042 DALYs_(0,0)_
Remediation scenario	7078 DALYs _(3,1)_
If actual exposed population is 25 % less	38581 DALYs_(3,1)_
If actual exposed population is 25 % greater	64266 DALYs_(3,1)_

## Discussion

This study sought to characterize the number of years lost due to illness, disability, or early death from lead exposure in Argentina, Mexico, and Uruguay. Environmental levels of lead were characterized in those countries and DALYs were calculated based on estimated exposure. In total, an estimated 51,432 DALYs from a total of 316,703 people exposed to lead at 129 toxic waste sites were located throughout the study region. This translates to approximately .31 DALYs_(3,1)_ per person. The estimated burden of disease as a result of exposure to lead was approximately 0.12 % of DALYs for all causes as estimated by the WHO in Argentina, Mexico and Uruguay [29].

By quantifying disease burden from lead pollution through a DALY-based method developed by Chatham-Stephens et al., comparisons can be made to other public health threats and illnesses. The modeled burden of disease estimated for exposure to lead at screened sites is comparable to the burden resulting from more widely recognized public health issues such as Parkinson’s disease (52,800 DALYs), Acute Hepatitis B and C combined (43,300) and bladder cancer (59,500 DALYs) in the three countries analyzed [28, 40]. The estimated burden of disease due to lead exposure is also greater than estimates for all childhood-cluster diseases including pertussis, diphtheria, measles, and tetanus (9,100 DALYs) and multiple sclerosis (27,500 DALYs) [29]. A comparison of DALYs from lead exposure and other health outcomes in Argentina, Mexico and Uruguay can be seen in Table 6, though it must be reiterated that DALYs from lead exposure are estimated rather than empirical.

**Table 6:** DALY Comparisons by Health Outcome

Selected Outcomes and Exposures	Total DALYs
Leishmaniasis	2800
Childhood-cluster Diseases^a^	9100
Multiple Sclerosis	27500
Chlamydia	39100
Acute Hepatitis B and C	43300
Lead Exposure (Modeled)	**51432**
Parkinson’s Disease	52800
Bladder Cancer	59500
Melanoma and Skin Cancers	63800
Tuberculosis	141500
Asthma	295700
Diarrheal Disease	375100
HIV	469100
Respiratory Infections^b^	153500
Diabetes Mellitus	3102600

DALYs 2012 estimates (WHO, 2014)

Bold data are based on our modeled estimate using a method developed by Chatham-Stephens et al

^a^Childhood-cluster diseases include pertussis, diphtheria, measles, and tetanus

^b^Respiratory infections includes lower respiratory infections, upper respiratory infections, and otitis media

The ingestion and inhalation of lead contaminated soil and dust was the main exposure pathway in the data analyzed. Biomarkers (blood lead levels) were used in the calculation of disease burden for lead exposure in Mexico and Uruguay, accounting for 47.4 % of the DALYs estimated in those countries.

Mexico is the fourth-largest producer of lead worldwide, with 222,000 metric tons generated in 2012 and a continually increasing output [41]. Sites in Mexico included in the lead exposure analysis were currently or previously involved with production of earthenware with leaded glaze (*n =* 31), mining operations (*n* = 22), smelting activities (*n* = 3), used lead-acid battery recycling (*n* = 1) and manufacturing (*n* = 5).

If BLLs were adjusted to below the “actionable” limit recommended by the CDC (5 ug/dL), an estimated 24,281 DALYs in Mexico (23,342 DALYs) and Uruguay (939 DALYs) could be eliminated. Such interventions include the introduction of lead-free glaze in ceramic ware, legislation to regulate battery-recycling, reduction of lead dust in homes, education about the health effects resulting from Pb exposure, as well as continued monitoring of BLLs. In comparison with the initial DALYs_(3,1)_ estimate, 44,354 DALYs_(3,1)_ could be eliminated if these sites were remediated. Despite producing lead in smaller quantities, exposure contributed significantly to disease burden in both Argentina (83,700 metric tons from primary and secondary lead smelting in 2013, 4,061 DALYs_(3,1)_) and Uruguay (no lead production data available, 2,051 DALYs_(3,1)_) [42].

A previous burden of disease study by Chatham-Stephens et al. found 54,432 DALYs attributable to lead exposure in India (*n* = 24), 78,982 DALYs in Indonesia (*n* = 28) and 394,084 DALYs in the Philippines (*n* = 27). While these estimates are larger than the estimated 45,321 DALYs attributable to lead-contaminated sites in Mexico (*n* = 62), an estimated 0.41 DALYs per person resulted from lead exposure at these sites, higher than previous estimates for India (0.21 DALYs per person), Indonesia (0.21 DALYs per person) and the Philippines (0.30 DALYs per person). In the same study, 0.10 DALYs per person were estimated for exposure to eight chemicals in India, Indonesia, and the Philippines (mean population at risk of exposure per site = 23,079) [4], while an estimated .31 DALYs_(3,1)_ per person occurred due to exposure to lead in Argentina, Mexico and Uruguay (mean population at risk of exposure per site = 2455). This higher average DALY per person was likely a result of a smaller population at risk and higher lead concentrations found in the three countries in this review.

A number of limitations for the calculation of disease burden should be noted. One such limitation has to do with extrapolation from a limited number of samples. The TSIP assessment process relies on minimal environmental sampling, composed of targeted and composited samples. The methodology was developed for screening purposes and is insufficient to fully characterize health risks at a site. As a result, the estimates here are necessarily indicative rather than definitive in nature.

A second significant limitation has to do with the limited number of sites captured by the TSIP. The number utilized here, 129, is very likely a significant undercount of the total number. Future efforts might endeavor to document additional sites or develop a robust methodology for modeling what that number might be.

A final limitation is the singular focus on lead. TSIP site investigators collect data for a range of pollutants including arsenic, hexavalent chromium, mercury, pesticides and particulate matter contributing to air pollution. However, these analyzed samples were too few in number to generate an accurate burden of disease estimate. In order for this exercise to be repeated with other pollutants, both data collection and site identification need to be improved. The use of mercury in artisanal small-scale gold mining (ASGM), for example, is a known threat to public health in Latin America, and future site investigations must continue to identify sites of mercury exposure [43]. As the analysis was solely focused on exposure to lead, it is likely that the burden of disease resulting from exposure to toxic pollution is largely underestimated.

## Conclusion

Intervention and remediation programs must focus on lead-contaminated sites in Argentina, Mexico and Uruguay as exposure to lead continues to contribute a significant disease burden for the population in these countries. An estimated 316,703 persons are subject to lead exposure at screened sites in these countries, resulting in 51,432 DALYs_(3,1)_. However, site investigations and efforts to estimate the burden of disease caused by pollution must continue to incorporate threats from exposure to mercury, arsenic, hexavalent chromium, pesticides, air pollution and other contaminants. Future studies should attempt to extrapolate these estimates to unscreened sites in an effort to approximate a more accurate burden of disease. This larger estimate is likely to be comparable with the burden of disease resulting from myriad chronic illnesses, and may be used as a tool to generate support and funding for the remediation of toxic waste sites in these countries and others. While the three countries of study have protocols in place to monitor children’s BLLs and reduce lead exposure, programs to regulate ULAB recycling exist only in Argentina, and regulations limiting the content of residential paint exist in only Argentina and Uruguay. Efforts to reduce the burden of disease resulting from lead exposure such as these and others must be implemented in all countries to adequately reduce the burden of disease from lead exposure.

## Abbreviations

ALM, Adult lead methodology; ASGM, Artisanal small-scale gold mining; BLL, Blood lead level; CDC, U.S. Centers for Disease Control; CSM, Conceptual Site Model; DALY, Disability-adjusted life year; DW, Disability weight; EC, European Commission; IEUBK, Integrated Exposure, Uptake and Biokinetic model; LMICs, Low- and middle-income countries; MMR, Mild mental retardation; TSIP, Toxic Sites Identification Program; ULAB, Used lead-acid battery; UNIDO, United Nations Development Organization; USEPA, United States Environmental Protection Agency; WHO, World Health Organization; YLD, Years lived with disability; YLL, Years of life lost
